# Reduction in antibiotic prescribing for respiratory tract infections in Swedish primary care- a retrospective study of electronic patient records

**DOI:** 10.1186/s12879-016-2018-9

**Published:** 2016-11-25

**Authors:** Mia Tyrstrup, Anders Beckman, Sigvard Mölstad, Sven Engström, Christina Lannering, Eva Melander, Katarina Hedin

**Affiliations:** 1 Department of Clinical Sciences, Family Medicine, Lund University, Malmö, Sweden; 2 Unit of Research and Development in Primary Care, Jönköping, Sweden; 3 Department of Infection Control, Malmö, Skåne County Sweden; 4 Department of translational medicine, Lund University, Malmö, Sweden; 5 Department of Research and Development, Region Kronoberg, Växjö, Sweden

**Keywords:** Antibiotic prescribing, Electronic patient records, Family practice, General practice, Primary healthcare, Respiratory tract infections

## Abstract

**Background:**

Swedish studies on antibiotic use in primary care have been based on one-week registrations of infections. In order to study adherence to guidelines, analyses based on large databases that provide information on diagnosis linked prescriptions, are needed. This study describes trends in management of infections in Swedish primary care particularly with regards to antibiotic prescribing and adherence to national guidelines.

**Methods:**

A descriptive study of Sweden’s largest database regarding diagnosis linked antibiotic prescription data, the Primary care Record of Infections in Sweden (PRIS), for the years 2008, 2010 and 2013.

**Results:**

Although the consultation rate for all infections remained around 30% each year, antibiotic prescribing rates decreased significantly over the years from 53.7% in 2008, to 45.5% in 2010, to 38.6% in 2013 (*p* = .032). The antibiotic prescribing rate for respiratory tract infections (RTIs) decreased from 40.5% in 2008 to 24.9% in 2013 while those for urinary tract infections and skin and soft tissue infections were unchanged. For most RTI diagnoses there was a decrease in prescription rate from 2008 to 2013, particularly for the age group 0–6 years. Phenoxymethylpenicillin (PcV) was the antibiotic most often prescribed, followed by tetracycline. Tonsillitis and acute otitis media were the two RTI diagnoses with the highest number of prescriptions per 1000 patient years (PY). For these diagnoses an increase in adherence to national guidelines was seen, with regards to treatment frequency, choice of antibiotics and use of rapid antigen detection test. The frequency in antibiotic prescribing varied greatly between different Primary Healthcare Centres (PHCCs).

**Conclusion:**

Falling numbers of consultations and decreased antibiotic prescription rates for RTIs have reduced the antibiotic use in Swedish primary care substantially. Overprescribing of antibiotics could still be suspected due to large variability in prescribing frequency, especially for acute bronchitis and sinusitis. Continuous evaluation of diagnosis linked prescribing data and feedback to doctors is essential in order to achieve a more prudent antibiotic use.

**Electronic supplementary material:**

The online version of this article (doi:10.1186/s12879-016-2018-9) contains supplementary material, which is available to authorized users.

## Background

Antibiotic use is associated with the development of antimicrobial resistance [1–6]. It has been estimated that 25,000 people die in Europe each year due to resistant bacteria and there are substantial costs associated with prolonged medical treatment time [7]. Negative effects on the environment are also seen, as high levels of resistant bacteria have been found in rivers contaminated with antibiotic substances [8–11]. Together with agriculture and animal production for food, the medical profession has a great responsibility to reduce the antibiotic consumption globally [12].

The frequency of antibiotic prescribing varies greatly between countries in Europe [13–17]. In Sweden 90% of the antibiotics are prescribed in out patient care, of which some 65% are prescribed in primary care, 25% in hospital outpatient department and outpatient department other than primary care, 8% in dental care.

Respiratory tract infections (RTIs) represent the majority (50–60%) of all infections in Swedish primary care and therefore are at focus for efforts to optimise antibiotic prescribing [18, 19] Media campaigns to the public and structured implementation of new guidelines regarding RTIs have been performed on a national basis. Many years of structured work by the Swedish strategic programme against antibiotic resistance (Strama) has significantly reduced the antibiotic use over the last decade [18, 20]. However, the variation in antibiotic prescribing rates between regions and between different practices indicates that there is still probable overprescribing [21, 22].

Within the last ten years, national guidelines regarding Lower Respiratory Tract infections (LRTIs), acute otitis media (AOM) and tonsillitis have been revised and all recommend a more restrictive use of antibiotics than earlier versions. The guidelines for LRTIs were revised in 2007 and they recommend Phenoxymethylpenicillin (PcV) as first line treatment for pneumonia and to abstain from antibiotics in acute bronchitis. When in diagnostic uncertainty, a C-reactive protein (CRP) test could be used. Guidelines for AOM (revised in 2010) recommend wait and see for three days and no immediate antibiotic treatment in children 1–12 years of age without complicating factors. The guideline for tonsillitis (updated in 2012) require the presence of 3 out of 4 Centor criteria (fever ≥38.5 tender cervical adenopathy, purulent tonsils and the absence of cough) and a positive rapid antigen detection test for Beta-haemolytic Streptococcus group A (RADT-Strep-A) before antibiotic treatment is initiated.

Gathering and analysing data on management of patients with infections in primary care forms an essential knowledge-base in order to identify new strategies for prudent antibiotic use. In Sweden, prudent use of antibiotics is defined as prescribing in adherence with current guidelines.

Previous Swedish studies on antibiotic use in primary care have been based on one-week registrations of infectious disease and therefore have included few data [23, 24]. Data from electronic medical records have provided valuable information on antibiotic prescription for upper respiratory tract infections in Sweden [25, 26]. In order to study adherence to guidelines, analyses based on large databases that provide information on diagnosis linked prescriptions, are needed in Sweden.

In 2007 the Primary care Record of Infections in Sweden (PRIS) was introduced. PHCCs were invited to participate on a yearly basis and contribute with data on consultations with an infectious diagnosis and all antibiotic prescriptions. In PRIS the majority of antibiotic prescriptions can be linked to a particular diagnosis, as can information on age, gender, results of CRP and RADT-Strep-A, if applicable, for each patient encounter. Preliminary results have shown a very variable prescription rate as well as variable adherence to guidelines [18, 27].

The aim of this study was to describe how antimicrobials were used in Swedish primary care, 2008–2013. We aim to identify trends over time, particularly for respiratory tract infections with regard to antibiotic prescriptions and adherence to national guidelines.

## Methods

This retrospective, descriptive study was based on the information retrieved from the PRIS register, from the years 2008, 2010 and 2013.

### The study population

The number of Primary Healthcare Centres (PHCC) contributing data was 47 in 2008, 58 in 2010 and 88 in 2013. In Sweden there were about 1200 PHCCs in total in 2010. In a typical PHCC in Sweden there are about 3–10 General Practitioners working. Together, the PHCCs represented in PRIS, served 460,529 registered patients in 2008 and 785,070 in 2013, of all ages, in both urban and rural areas of Sweden. Accordingly, the number of registered patients in 2013 represented 8% of Sweden’s total population of 9,644,864 inhabitants in 2013 [28]. Due to the large number of registered patients in the material the number of patients in each age group was assumed to correspond to the total inhabitants of Sweden as of December 31, for respective year [28].

For each year all registered contacts were traced either due to the presence of an infectious diagnosis according to the International Classification of Disease and Related Health Problems- Tenth Revision (ICD-10), introduced by the WHO, or because an antibiotic prescription by Anatomical Therapeutic Chemical Classification (ATC)-code had been registered. The ATC coding system is used to classify drugs to facilitate studies of drug use in Sweden. With each registered encounter information on the patient’s age, sex and if applicable laboratory testing and antibiotic prescription was recorded. Common near patient tests in Sweden are CRP and RADT-Strep-A.

Some patient encounters had more than one registered diagnosis and sometimes an extra symptom diagnosis had been added, such as cough. The diagnoses in PRIS have therefore been ranked, so that the highest ranked is the diagnosis most likely to cause an antibiotic prescription according to clinical practice. Of the most common RTI diagnoses,, pneumonia has the highest rank, followed by AOM, tonsillitis, sinusitis, acute bronchitis, pharyngitis, common cold and symptom diagnoses like cough. If a contact had generated more than one diagnosis only the highest ranked was retained. The same ranking system was used all years.

### Data processing

The different diagnoses were divided into four groups according to type of infection; respiratory tract including ear infections (RTI), urinary tract (UTI), skin and soft tissue (SST) and other infections. The latter group consisted of diagnoses found in Chapter A and B of the ICD-code system, including relatively few patients, and some other diagnoses such as diverticulitis, gynaecological-, and sexual transmitted infections.

RTIs were further divided into upper RTIs (URTIs) and lower RTIs (LRTIs). URTIs were organised into groups that share similarities in clinical practice or national guidelines, such as common cold, sore throat, acute otitis media (AOM) and sinusitis. The group of sore throat consisted mainly of tonsillitis but also acute pharyngitis, mononucleosis, quinsy and scarlet fever. AOM was divided into age groups, due to specific Swedish national guidelines regarding children 1–12 years old.

The LRTIs were divided into chronic obstructive disease (COPD), cough, acute bronchitis, pneumonia and influenza as these groups differ with regard to recommended treatment. Nonspecific chronic bronchitis was included in the COPD.

The consultation and antibiotic prescription rates regarding RTIs in Table 2 and the corresponding figures for total consultation and antibiotic prescription in Table 3 differ slightly. This is because there were some differences as to which diagnostic codes were included in each calculation. The arrangement of diagnoses in Table 2 was done in the initial set up of the variables in PRIS. In Table 3 the inclusion of diagnoses was made later, based on a selection of diagnostic codes from ICD-10. The group “no infectious diagnosis” in Table 2 refers to antibiotic prescriptions with non-infectious diagnosis or no diagnosis registered.

In order to calculate the number of antibiotic prescriptions per 1000 patient year (PY) each PHCC provided information on the number of registered patients at their clinic, calculated as a mean for each year. PY refers to the number of registered patients in the participating PHCCs, per year.

In Table 4 variability in antibiotic prescription rate for five common diagnoses (AOM, sinusitis, tonsillitis, acute bronchitis and pneumonia) is presented. Four of the five diagnoses also appear in Table 3, but with different values for antibiotic prescription rates. The figures in Table 4 were calculated on PHCC level and the figures regarding antibiotic prescription rate in Table 3 referred to an individual level.

Data from the 37 PHCCs who participated all three years, with approximately 380,000 registered patients, was compared to that of all PHCCs. Percentage of consultations for infections, prescribed antibiotics and number of prescription per 1000 PY were calculated (Additional file 1).

### Statistical method

All analyses were performed using Excel (Microsoft. Microsoft Excel. Redmond. Washington: Microsoft, 2010. Computer Software) and SPSS (IBM Corp. Released 2013. IBM SPSS Statistics for Windows, version 22.0.Armonk, NY: IBM Corp).

For descriptive statistics, proportions and standard deviations were used. Comparisons between proportions of categorical variables in more than two independent groups were performed with the Chi-square test for trend, using an on-line calculator, http://epitools.ausvet.com.au. *P*-values ≤ .05 were considered statistically significant.

## Results

The number of PHCCs providing data to the PRIS record increased over the three years we studied. The population they served increased accordingly, as did the number of consultations for all causes. The percentage of consultations due to infection remained about 30% for each year. (Table 1) 60% of the consultations for infections were made by women each year. The total antibiotic prescription rate decreased significantly from 53.7% in 2008 and 45.5% in 2010 to 38.6% in 2013 (*p* = .032). (Table 1) The prescription rates for the 37 PHCCs that participated all years did not differ from the total regarding proportion of consultations that rendered an antibiotic prescription or in the number of prescriptions per 1000 PY (Additional file 1).

**Table 1:** Characteristics of the infections disease dataset

	2008	2010	2013
Number of Primary Healthcare Centres (PHCC)	47	58	88
Number of patient years (PY)	460 529	556 192	785 070
Number of consultations (all causes)	662 184	809 964	1 085 829
Consultations due to infections (percentage of all consultations)	210 388 (31,8%)	245 344 (30,3%)	318 976 (29,4%)
Prescribing rate	53,7%	45,5%	38,6%

### Consultations for all infections

RTIs, including AOM, was the most common group of infections, representing more than half of the consultations and resulted in the highest number of prescriptions per 1000 PY. UTIs and SST infections were the second and third most common reason for consultation. The prescribing of antibiotics per 1000 PY decreased significantly for RTIs, but remained unchanged for UTIs and SST infections (Table 2).

**Table 2:** Consultations and antibiotic prescribing according to type of infection per 1000 person years (PY), years 2008, 2010 and 2013

	2008			2010			2013		
	Consultations per 1000 PY	Share of all infection consultations (%)	Antibiotic prescriptions per 1000 PY	Consultations per 1000 PY	Share of all infection consultations (%)	Antibiotic prescriptions per 1000 PY	Consultations per 1000 PY	Share of all infection consultations (%)	Antibiotic prescriptions per 1000 PY
Respiratory tract, incl AOM^a^	251	54,9	102	254	57,5	84	227	55,8	56
Skin and soft tissue	49	10,8	31	53	12,0	29	64	15,7	30
Urinary tract	45	9,9	36	50	11,2	38	48	11,9	36
Other infections^b^	36	7,9	3	36	8,1	3	33	8,2	2
No infectious diagnosis^c^	76	16,6	74	49	11,2	48	34	8,3	32
All	457	100	245	441	100	201	406	100	157

^a^Diagnostic group excluding diagnoses in chapter 1 of ICD-code system

^b^Diverticulitis, STI, gynaecological- and viral infections

^c^Prescriptions with non-infectious diagnosis or no diagnosis registered

The frequency of prescriptions without a diagnosis decreased from 22% in 2008 to 13% in 2013. Antibiotic prescriptions with no registered diagnosis could, due to the type of antibiotic prescribed, be allocated to probable RTIs (PcV, amoxicillin, cephalosporin, doxycycline and macrolides) in 53% (2008), 46% (2010), 41% (2013) of the cases and to UTIs (pivmecillinam, nitrofurantoin, trimethoprim and quinolones) in 28% (2008), 29% (2010) and 40% (2013). SST type antibiotics (betalactam resistant penicillin and clindamycine) represented about 20% of the prescriptions with no diagnosis in all three years.

### Consultations and antibiotic prescriptions for RTIs

**Fig. 1:**
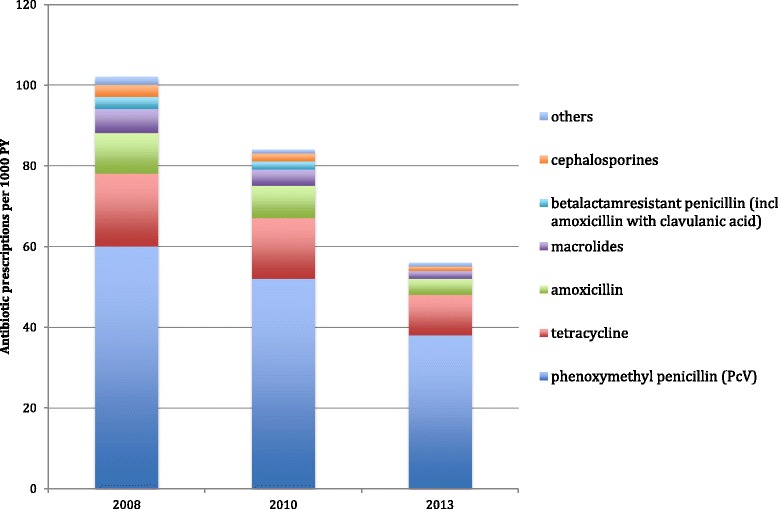
Trends of antibiotic subclasses for treatment of RTIs in the years 2008, 2010 and 2013

PcV was the most common antibiotic prescribed for RTIs followed by tetracycline. The proportion of PcV increased and all other antibiotic subclasses decreased from 2008 to 2013 (Fig. 1).

The number of consultations as well as the number of antibiotic prescriptions per 1000 PY for the diagnoses common cold, sore throat, AOM and sinusitis decreased simultaneously over the years (Table 3).

**Table 3:** Consultations and antibiotic prescriptions per 1000 patient years (PY) in different RTI diagnoses, years 2008, 2010, 2013

		2008			2010			2013		
		Consultations per 1000 PY	Antibiotic prescription per 1000 PY	Prescribing rate per diagnosis, (%)	Consultations per 1000 PY	Antibiotic prescription per 1000 PY	Prescribing rate per diagnosis, (%)	Consultations per 1000 PY	Antibiotic prescription per 1000 PY	Prescribing rate per diagnosis, (%)
Upper respiratory tract infections	Common cold	103	12	11,8	101	8	7,8	86	4	4,5
	Sore throat^a^	42	28	67,7	38	23	61,4	29	18	59,7
	AOM^b^	27	23	84,5	24	20	80,6	16	12	74,9
	Sinusitis	17	14	81,0	15	11	70,9	12	7	60,6
Lower respiratory tract infections	Cough	24	2	8,3	30	2	6,5	28	1	4,0
	COPD	11	2	14,5	17	2	11,1	28	2	7,8
	Acute bronchitis	22	13	59,7	22	10	45,4	18	5	26,2
	Pneumonia	10	7	66,2	11	7	61,9	11	7	62,5
	Influenza	2	0,1	6,3	1	0,1	7,4	4	0,1	3,3
Total^c^		258	101		259	83		232	55	

^a^Sore throat comprises the diagnosis tonsillitis, pharyngitis and mononucleosis, quinsy and scarlet fever.^b^Acute Otitis Media (AOM) ^c^Total values for RTIs add up to higher values than RTIs in Table 2 due to inclusion of some diagnoses from chapter 1 of ICD-code system

Sore throat was comprised of consultations labelled as tonsillitis in about two thirds of the cases, pharyngitis in one third, and one to two percentages each of mononucleosis, scarlet fever and quinsy. This distribution remained the same over the three years.

Tonsillitis was the condition with the highest prescribing rate among the respiratory tract infections. PcV was used in most cases of tonsillitis, with an increasing frequency over the years. The number of patients with tonsillitis who were prescribed antibiotics, without having been tested with RADT-Strep-A decreased from 39.7% in 2008 to 26.9% in 2013. The patients who were prescribed antibiotics, despite a negative RADT-Strep-A test also decreased from 24.7% in 2008 to 13.1% in 2013.

**Fig. 2:**
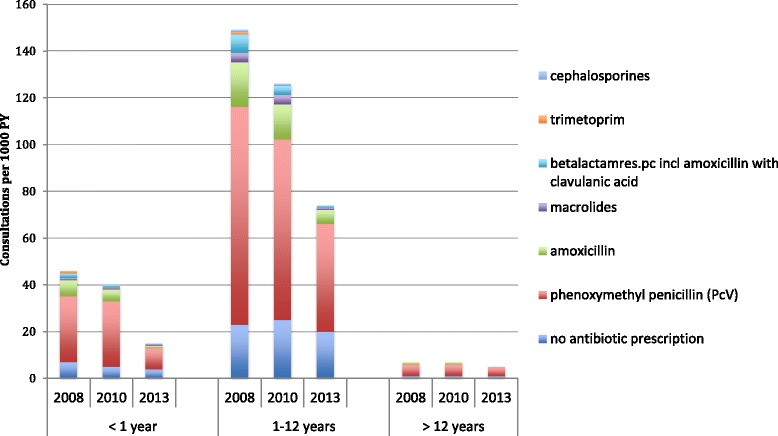
Trends for treatment of AOM according to age group in the years 2008, 2010 and 2013

The number of antibiotic prescriptions per 1000 PY for AOM was halved between 2008 and 2013. Children 1–12 years of age comprised about two thirds of the cases of AOM all three years. (Fig. 2) The fraction given no antibiotics increased significantly from 15.4% in 2008 to 27.0% in 2013 (p = .041) and the percentage of patients treated with PcV increased from 73.6% in 2008 to 84.5% in 2013. Among children below 1 year of age the number of patients diagnosed with AOM decreased significantly as did the prescribing of antibiotics (Fig. 2).

The consultation rate for pneumonia remained stable over the years. Pneumonia was most often treated with PcV (53.8%). Although the consultation rate for acute bronchitis remained stable from 2008 to 2013, the antibiotic prescription rate decreased from 59.7% in 2008 to 26.2% in 2013. The most commonly used antibiotic for acute bronchitis was tetracycline (54.1%).

### Variability in prescribing between PHCCs

The antibiotic prescribing rate per diagnostic group varied between different PHCC. The largest difference between the lowest and the highest prescribing PHCC was found for the diagnoses acute bronchitis and sinusitis. The mean antibiotic prescription rate per diagnoses per PHCC has decreased for all diagnoses between 2008 and 2013, but the standard deviation (SD) as an indicator for variability has increased for all diagnoses except pneumonia (Table 4).

**Table 4:** Variability in antibiotic prescription rate between PHCCs, for RTIs commonly treated with antibiotics

	2008	2010	2013
	Prescription rate % (SD^a^)	Prescription rate % (SD^a^)	Prescription rate % (SD^a^)
AOM^b^	84.5 (7.9)	79.3 (8.1)	74.4 (8.5)
Sinusitis	80.4 (8.7)	70.6 (12.1)	62.5 (12.7)
Tonsillitis	88.4 (8.0)	83.6 (9.3)	80.0 (10.1)
Acute bronchitis	60.1 (15.5)	45.6 (17.3)	28.2 (17.4)
Pneumonia	66.1 (12.1)	61.1 (12.0)	63.0 (9.7)

^a^Standard Deviation (SD)

^b^Acute Otitis Media (AOM)

### Use of CRP

CRP was analysed in almost one fourth of all consultations throughout the three years. Almost one third (28.5%) of the patients with sore throat diagnosed in 2013 had a CRP taken. Among the lower respiratory tract infections CRP was used most often among patients diagnosed with pneumonia (60.4%) and acute bronchitis (49.4%).

### Consultation and prescribing for RTIs in different age groups

The consultation rate decreased for all age groups, except for 65 years and older, from 2008 to 2013. (Table 5) The prescribing of antibiotics decreased in all age groups, with the largest change (54%) in the age group 0–6 years, from 297 to 136 prescriptions per 1000 PY. PcV was the most common antibiotic in all age groups except in the age 65 years and older, where tetracycline was slightly more common.

**Table 5:** Consultations and antibiotic prescriptions per 1000 patient years (PY) for RTIs in 2008, 2010 and 2013, for different age groups

	2008		2010		2013	
Age-Group (years)	Consultations per 1000 PY	Antibiotic prescription per 1000 PY	Consultations per 1000 PY	Antibiotic prescription per 1000 PY	Consultations per 1000 PY	Antibiotic prescription per 1000 PY
0–6	712	297	703	254	467	136
7–18	247	109	251	91	171	62
19–64	204	84	200	67	181	47
65-	206	68	228	57	280	48
Total	251	102	254	84	229	56

## Discussion

### Main findings

The antibiotic prescribing rate for RTIs decreased from 40.5% in 2008 to 24.9% in 2013, while those for UTIs and SST infections were unchanged. All the diagnoses regarding URTIs showed a decrease in both consultation and prescription rate during the years 2008–2013. Figures suggested increased adherence to guidelines for AOM, LRTIs and tonsillitis.

We noted substantial reduction in antibiotic prescribing for acute bronchitis. Increased variability in prescribing rates between different PHCCs was found, particularly for the diagnoses acute bronchitis and sinusitis.

### Findings in relation to current guidelines

Overall these findings indicate an increased adherence to guidelines over time. This is reflected in the reduction in the numbers of patients diagnosed with AOM, the increased prescription of PcV in children and the increased rate of treatment with no-antibiotic in children (Fig. 2), and the decrease in antibiotics for tonsillitis and pharyngitis. While it should be noted that the introduction of the pneumococcal vaccine in the general vaccination program in Sweden in 2008–2009 may also have played a part in the reduction of AOM in the age group <1 year. [29, 30], taken together these findings suggest an increased adherence to diagnosis and treatment guidelines.

Furthermore, the antibiotic prescribing rate for acute bronchitis decreased substantially and is approaching the recommended level of less than 20% according to Swedish College of General Practice. The consultation and prescription rates for pneumonia were constant over the three years. Thus, there does not seem to have been a shift in choice of diagnosis from acute bronchitis to pneumonia to explain the decrease in use of antibiotics for acute bronchitis. For all RTIs, except acute bronchitis and COPD the most commonly used substance was PcV, which is in accordance with national guidelines.

However, adherence to guidelines can still be improved since many of the patients treated with antibiotics, with the diagnosis tonsillitis, had no RADT-Strep-A taken or a negative test. Swedish interview studies have indicated that high prescribing doctors were not aware of or not updated on the national guidelines for tonsillitis [31], which may explain the current findings. Finally, excessive use of CRP as a diagnostic tool, was noted, particularly in association with common cold and sore throat, which is not according to guidelines.

Also the increased variability in antibiotic prescription rate per diagnoses between different PHCCs (Table 4) suggests inappropriate antibiotic prescribing. Further analyses as to what factors are important for changing prescribing behaviours are needed.

### Previous studies

Our findings indicate a decrease in antibiotic prescribing rate for RTIs. This is in line with previous Swedish studies based on electronic patient records [25, 26] and a diagnosis- prescribing survey [23] performed in the early 2000.

The continuous reduction in consultation rates seen in previous studies as well as in ours could be an effect of the arising debate in media and among the public with a more critical view of the use of antibiotics, which started in the early 90’s. In addition, an increased focus on the health care level with campaigns to reduce antibiotic prescribing by educational efforts towards health professionals and national targets for good practice, may have played a part in the reduction in consultation rates as well as antibiotic prescription rates, as was noted in 2008–2013 [20].

Studies based on large diagnosis linked prescription registers can, among other countries, be found in the United Kingdom (UK) and the Netherlands. In contrast to Sweden, the antibiotic prescribing for coughs/cold is reported to have increased in the UK over the years 1995–2011 despite implementation of national guidelines [32]. The increase seem to be due to an increased consultation incidence for coughs/cold in the UK, which was not seen in Sweden. A possible explanation is the gate-keeping function of the nurse triage. As a rule, to book an appointment with a GP in a Swedish PHCC the patient need to speak to a nurse first. The nurses’ triage is performed according to current guidelines and in many cases you will be given enough advice by the nurses at this level and will not need to see the GP. In addition, in Sweden most patients do not need a sick-leave note the first week of illness for themselves or for a sick child. This may explain why numbers of consultations for RTIs are low in Sweden compared to other countries [32, 33].

In the Netherlands antibiotic prescribing has been rather stable and not decreased between 2007 and 2010 [33]. Antibiotics were prescribed in 26% of infectious disease episode in the Netherlands in 2010 and in 45,5% the same year in Sweden. However, the Dutch recorded more infectious episodes per patient year (1065) compared to what was found in our material (441) for 2010. Again, the gate-keeping function may play a role to keep consultation rates low in Sweden. Similarly, for AOM, the antibiotic prescription rate in the Netherlands was low (45.8%) compared to Sweden (80.6%) in 2010, but since the consultation for AOM per 1000 PY was lower in Sweden (24.3) compared to the Netherlands (38.2) the antibiotic prescription per 1000 PY was rather similar, 20 for Sweden and 17 for the Netherlands. (80.6% of 24.3 = 20 and 45.8% of 38.2 = 17).

Sore throat, including tonsillitis and pharyngitis, is noted to have similar treatment frequencies in the UK (62%), the United States (60%) and Sweden (60%) but a little lower in the Netherlands (55%) [32–34]^,^ The reason for the relatively high consultation rate for sore throat in Sweden is believed to be associated with the high availability of the RADT-Strep A at every PHCC, as well as other factors.

### Strength and limitations

The PRIS database contains the largest number of patient registrations with diagnosis linked data on antibiotic prescribing in primary care in Sweden. The PHCCs represented in the database are participating on a voluntary basis, which might cause a selection of more motivated PHCCs and hence, better prescribing rates. On the other hand, the fact that in some of the geographic areas, all of the PHCCs participate contradicts a selection bias. In addition, private and public PHCCs in both rural and urban areas are represented. The number of participating PHCCs increased from 2008 to 2013 and accordingly, the number of consultations, but the percentage related to an infection remained constant (30%), which reduces the likelihood of a very strong selection bias. It can be argued that all calculations should be based only on the PHCCs participating all years, however that would make some of the data less reliable, for example AOM and age groups. We therefore compared the prescription rate and number of prescriptions per 1000 PY for the 37 PHCCs that participated all years with the total and did not find any differences (Additional file 1). In addition, the antibiotic prescription rates for UTIs and SST infections remained unchanged while a reduction was seen for RTIs. This reduction was in line with the revised and implemented guidelines for RTIs (AOM, LRTIs and tonsillitis) during the study period.

According to the Public Health Agency of Sweden the mean total antibiotic prescriptions per 1000 inhabitants in Sweden were 443 in 2008, 406 in 2010 and 343 in 2013 [18]. So, the national data on sale show a reduction of 100 prescriptions per year between 2008 and 2013 and in our study we found a decrease of 88 prescriptions (from 245 to 157). This indicates that our results are in line with trends in national data on antibiotic sales and that most of the reduction in antibiotic use may have taken place in primary care.

A limitation is that no visits after office-hours were included in the register, which could cause an underestimation of the frequency of infections as well as of antibiotic prescribing. Control visits could not be distinguished from a first visit, which might cause overestimation of the number of certain diagnoses such as pneumonia and AOM and hence also underestimation of the percentage of antibiotic prescribing for these diagnoses.

Another limitation is that for 13.5% of the prescriptions there was not a linked diagnosis. The majority (2/3) of these antibiotics were prescribed without a patient visit. Possible explanations are prescriptions for patients at nursing homes, from consultations by telephone or prescriptions being registered later after receiving results from cultures or x-rays.

## Conclusion and future directions

This study supports that primary healthcare in Sweden has succeeded to improve prescribing of antibiotics in accordance to national guidelines. Both consultations and and antibiotic prescribing for RTIs have decreased. The significant decrease in prescribing rate for AOM and tonsillitis indicate that updating and implementing national guidelines is an important tool to reduce antibiotic use.

There are still large variations in prescribing frequencies between different PHCCs, indicating that irrational antibiotic prescribing still exists. Continuous evaluation of diagnosis linked prescribing data and feedback to doctors is essential in order to make changes towards more prudent antibiotic use.

## Additional file

Additional file 1: Sensitivity analysis. (DOCX 18 kb)
