# Supplementary material for: Reduction in antibiotic prescribing for respiratory tract infections in Swedish primary care- a retrospective study of electronic patient records
Source: BMC Infect Dis. 2016 Nov 25;16:709. doi: 10.1186/s12879-016-2018-9 (PMC5124268; doi:10.1186/s12879-016-2018-9)
Supplement: Additional file 1: — Sensitivity analysis. (DOCX 18 kb) [file 12879_2016_2018_MOESM1_ESM.docx]

# Additional file 1; sensitivity analysis

Table S1. Characteristics of the infectious disease dataset

|  | 2008 | | 2013 | |
| --- | --- | --- | --- | --- |
|  |  |  |  |  |
|  | All | Constant 37 | All | Constant 37 |
| Number of Primary Healthcare Centres (PHCC) | 47 | 37 | 88 | 37 |
| Number of patient years (PY) | 460 529 | 369 548 | 785 070 | 379 338 |
| Number of consultations (all causes) | 662 184 | 513 698 | 1 085 829 | 563 107 |
| Consultations due to infections (percentage of all consultations) | 210 388 (31.8%) | 162 890 (31.7%) | 318 976 (29.4%) | 153 193 (27.2%) |
| Prescribing rate | 53.7% | 51.8 | 38.6% | 38.9 |
| Prescriptions per 1000 patient years | 245 | 228 | 157 | 157 |

Consultation rates and antibiotic prescribing rates were calculated for all participating PHCCs and for the constant 37 PHCCs, participating all three years. Reductions in antibiotic prescribing rate for all PHCCs and for the constant 37 PHCCs were significant, p<0.01.

Table S2 Consultations and antibiotic prescribing according to type of infection per 1000 person years (PY), years 2008 and 2013

|  | 2008 | | | | | | 2013 | | | | | |
| --- | --- | --- | --- | --- | --- | --- | --- | --- | --- | --- | --- | --- |
|  | Consultations per 1000 PY | | Share of all infection consultations (%) | | Antibiotic prescriptions per 1000 PY | | Consultations per 1000 PY | | Share of all infection consultations (%) | | Antibiotic prescriptions per 1000 PY | |
|  | All | *Const.37* | All | *Const.37* | All | *Const.37* | All | *Const.37* | All | *Const.37* | All | *Const.37* |
| Respiratory tract, incl AOM* | 251 | *247* | 54.9 | *55.9* | 102 | *96* | 227 | *227* | 55.8 | *56.2* | 56 | *59* |
| Skin and soft tissue | 49 | *48* | 10.8 | *10.8* | 31 | *29* | 64 | *59* | 15.7 | *14.6* | 30 | *28* |
| Urinary tract | 45 | *42* | 9.9 | *9.8* | 36 | *36* | 48 | *47* | 11.9 | *11.7* | 36 | *34* |
|  | - | *-* | - |  | - |  | - | *-* | - |  | - |  |
| All | 457 | *441* | 100 | *100* | 245 | *228* | 406 | *404* | 100 | *100* | 157 | *157* |

Consultations and antibiotic prescriptions per 1000 patient per year (PY) were calculated for all participating PHCCs and for the constant 37 PHCCs participating all three years.
